# Perspectives of US women participating in a candidate PrEP study: adherence, acceptability and future use intentions

**DOI:** 10.1002/jia2.25247

**Published:** 2019-03-14

**Authors:** K Rivet Amico, Catalina Ramirez, Margaret R Caplan, Brooke EE Montgomery, Jennifer Stewart, Sally Hodder, Shobha Swaminathan, Jing Wang, Noshima Y Darden‐Tabb, Marybeth McCauley, Kenneth H Mayer, Timothy Wilkin, Raphael J Landovitz, Roy Gulick, Adaora A Adimora, HPTN 069/A5305 Study Team and HPTN Women at Risk Committee

**Affiliations:** ^1^ University of Michigan School of Public Health Ann Arbor MI USA; ^2^ University of North Carolina at Chapel Hill School of Medicine Chapel Hill NC USA; ^3^ David Geffen School of Medicine at UCLA Los Angeles CA USA; ^4^ University of Arkansas for Medical Sciences College of Public Health Little Rock AR USA; ^5^ Johns Hopkins School of Nursing Baltimore MD USA; ^6^ West Virginia University School of Medicine Morgantown WV USA; ^7^ West Virginia Clinical and Translational Science Institute Morgantown WV USA; ^8^ Rutgers New Jersey Medical School Newark NJ USA; ^9^ Fred Hutchinson Cancer Research Center Seattle WA USA; ^10^ FHI360 Washington DC USA; ^11^ Fenway Health and Harvard Medical School New York NY USA; ^12^ Weill Cornell Medicine New York NY USA

**Keywords:** PrEP, HIV prevention, women, United States, risk perception

## Abstract

**Introduction:**

Limited data exist on acceptability of candidate pre‐exposure prophylaxis (PrEP) regimens among US women. We evaluated PrEP experiences, attitudes and future use intentions among sexually active women who completed the US‐based HIV Prevention Trials Network 069/AIDS Clinical Trials Group 5305 study.

**Methods:**

Women participated in the study between March 2013 and November 2015. We analysed computer‐assisted self‐interview (CASI) surveys among 130 women and conducted in‐depth interviews among a subset of 26 women from three sites. Interviews were conducted in mid/late‐2015.

**Results:**

Most women (57%) reported very good/excellent PrEP adherence on CASI, although 21% acknowledged over‐reporting adherence at least some of the time. Commitment to preventing HIV infection, a sense of ownership of the study, and keeping pills stored in a visible location facilitated adherence. Adherence barriers included “simply forgetting” and being away from home. Most women interviewed did not intend to use PrEP in the future because of lack of perceived need due to their own (as opposed to their partners’) low‐risk behaviour and concerns about affordability – but not because of side effects or other characteristics of the regimens.

**Discussion:**

Improving HIV prevention options for US women will require access to affordable PrEP as well as expanding women's understanding of relationship‐ and community‐level factors that increase their risk of acquiring HIV.

## 1 Introduction

Although the number of new HIV infections in the United States (US) has declined over the past decade, as of 2015, women constituted almost one quarter (24%) of all people living with HIV 1. Until recently, HIV prevention strategies available to women at risk of heterosexual transmission were largely limited to approaches that required male partner cooperation (i.e. female or male condoms). Products that are safe, acceptable, discreet and can be controlled by women are needed. Oral pre‐exposure prophylaxis (PrEP) may be one such strategy. Randomized controlled trials (RCTs) have demonstrated that daily oral tenofovir disoproxil fumarate/emtricitabine (TDF/FTC), when used correctly and consistently, is highly effective for preventing HIV infection among men and transgender women who have sex with men (MSM) 2, 3, 4, HIV‐seronegative partners in serodiscordant relationships 5, and heterosexual men and women 6. However, two PrEP RCTs in sub‐Saharan Africa that exclusively enrolled women 7, 8 failed to demonstrate efficacy, largely due to study drug non‐adherence. A review of oral PrEP efficacy trials in women found that drug levels consistent with daily pill‐taking were associated with protection 7, suggesting that adherence is the critical factor influencing protective outcomes.

Adherence, as well as attitudes and beliefs about PrEP, among MSM in the US has received considerable attention in the literature 8, 9, 10, 11, 12, 13, 14, 15, 16, 17, 18, 19, 20, 21, 22, 23. However, few studies have been published regarding attitudes towards use of PrEP among women who have accumulated real experiences taking candidate PrEP products 24, 25, 26, 27. Moreover, few PrEP demonstration projects among US women have been planned 28, leaving a substantial at‐risk population in the US largely under represented. Despite PrEP's promise as an HIV prevention tool for US women, the lack of data on product acceptability and adherence in this population (especially in the setting of inconsistent adherence demonstrated in international clinical trials) may challenge its successful implementation for HIV prevention among women in the US.

In response to this paucity of data, we collected quantitative and qualitative data from women participating in HIV Prevention Trials Network (HPTN) 069/AIDS Clinical Trials Group (ACTG) 5305 study 29 to better understand reasons for adherence and/or non‐adherence to study drug, attitudes towards PrEP and intentions to use PrEP after the end of the study. Given the few PrEP trials involving US women and demonstration projects seeking to involve them, the results of this study may help develop approaches for supporting adherence in the rollout of current and future PrEP technologies.

## 2 Methods

### 2.1 Clinical trial

HPTN069/ACTG5305 was a prospective, randomized, double‐blinded, multisite, safety and tolerability study of four antiretroviral regimens for HIV PrEP: (1) maraviroc (MVC) alone; (2) MVC + emtricitabine (FTC); (3) MVC + tenofovir (TDF); and (4) TDF + FTC (control) conducted with women in the US between March 2013 and November 2015 29. As described in detail in the presentation of primary outcomes 29, study regimens consisted of three pills once‐daily: MVC 300 mg, FTC 200 mg and TDF 300 mg, with matching placebos. HIV‐seronegative women who reported a history of condomless vaginal or anal intercourse with >1 HIV‐seropositive male partner or man of unknown serostatus within 90 days, and had adequate safety laboratory parameters were enrolled at 12 US‐based study sites (Baltimore, MD; Boston, MA; Chapel Hill, NC; Cleveland, OH; Los Angeles, CA; Newark, NJ; New York City, NY; Philadelphia, PA; Pittsburgh, PA; San Juan, PR; Seattle, WA; and Washington, DC). Participants received randomized study regimens for 48 weeks with follow‐up visits at weeks 2, 4 and 8, and then every eight weeks through week 48. At each study visit, interval history, physical examination, safety laboratories, blood plasma for drug levels, HIV testing and adherence counselling were performed at all study visits. Electronic drug monitoring was used throughout the trial via a single pillbox (Wisepill™, Wisepill Technologies, Cape Town, South Africa) containing the three study medications.

The study protocol and the procedures for the qualitative substudy were approved by institutional review boards at the local institutions affiliated with each study site. A written informed consent was obtained from all participants prior to conduct of any study‐related activities.

### 2.2 Survey data

Detailed behavioural risk assessments and HIV risk perception were assessed via computer‐assisted self‐interview (CASI) at baseline and serially (every eight weeks). Attitudes towards study drug, barriers and facilitators to adherence, and intentions to use PrEP after the study's conclusion were measured at week 48. Adherence‐related facilitators and barriers were identified by women using a list where they could select all that applied.

We reviewed CASI data specific to beliefs, attitudes and adherence among the 130 women who were on study drug at week 48. As reported in the presentation of the primary outcomes for women in the parent study (HPTN069/ACTG5305) 29, of 188 women enrolled, 160 (85%) completed the study follow‐up, and of them, 30 participants had permanently discontinued the study regimen prior to week 48. Thus, we focused on CASI data from women with recent experiences with the study drugs (N = 130 women on study medication). Although the study used biological markers of adherence in a subset of women, the current evaluation focused on self‐report data and did not seek to distinguish between those with and without detectable drug at week 48.

#### 2.2.1 Analyses

Data analysis was performed for all enrolled women who completed the parent trial and had not discontinued their assigned study regimen. CASI data were analysed using SAS 9.4 (SAS Institute, Cary, NC). Descriptive statistics were performed to provide a demographic profile of study participants and summarize survey responses.

### 2.3 In‐depth interviews

A subset of 26 women from three sites (New Jersey, New York and North Carolina) were interviewed within two weeks of study completion (week 48). Convenience sampling was used, as the qualitative protocol was adopted late in the parent study and there was no opportunity to randomly select from the small pool of women who remained in the study. Site staff invited study participants to join the qualitative substudy as they completed their final study assessments. Interviews were conducted with a total of twenty‐six women, which included twenty‐three who were on study drug at the end of the study and three who had discontinued prior to study end. In contrast to CASI collected data, we retained the three who had discontinued study drug in the interview subset of women because interviewers could provide the opportunity to probe and unpack experiences with study drug over the course of the study.

Interview domains in the structured interview guide included experiences using a candidate PrEP regimen, attitudes towards the study drug, factors contributing to product adherence or non‐adherence, HIV risk perception and intention to use PrEP after study completion. Interviews were conducted by a trained interviewer identified at each site and lasted an average of 1.5 hours. Participants received $75 as compensation for their time and were reimbursed for transportation. All activities were approved by the Institutional Review Boards at Rutgers New Jersey Medical School (NJ), Weill Cornell Medicine (NY) and the University of North Carolina at Chapel Hill (NC).

#### 2.3.1 Analyses

Interviews were transcribed, coded and analysed in Dedoose 7.0.23 (SocioCultural Research Consultants, Los Angeles, CA) using a thematic framework analysis approach 30, 31, 32. Five trained analysts reviewed the transcribed interviews and applied coding “frames” based on the in‐depth interview (IDI) guide. Each frame was iteratively reviewed by several members of the coding team in order to identify and document key themes across the interview. Through this process, a codebook was developed with detailed and nuanced definitions of key themes, supporting themes and example quotes. Thematic codes were applied to transcripts using Dedoose. Intercoder agreement was assessed at various points in the analysis process. Coding discrepancies were discussed by the analysis team, the codebook revised accordingly and recoding performed when necessary to ensure consistent application of codes. To identify the most salient themes, code frequency reports and coded text reports were generated.

## 3 Results

### 3.1 Participant characteristics (Table 1)

**Table 1:** Characteristics among women participants in HPTN069/ACTG5305 who completed week 48 on study regimen

	All N = 130
Age	
Median (range)	39 (18 to 61)
Marital status	
Married/civil union	13 (10%)
Dating, living with partner	27 (21%)
Dating, not living with partner	15 (12%)
Single/divorced/widowed	73 (56%)
Other	2 (2%)
Employment status	
Full‐time employment	30 (23%)
Part‐time employment	24 (19%)
Not employed	76 (59%)
Education	
<High School	18 (14%)
High school graduate	46 (35%)
Vocational/technical school	8 (6%)
Some college or two‐year degree	34 (26%)
Finished college or graduate school	24 (19%)
Ethnicity – Latino	
Hispanic/Latino	17 (13%)
Race^a^	
Black or African American	83 (64%)
White	34 (26%)
Other	13 (10%)
Sexual behaviour at baseline	
Vaginal sex with a man in the past month	106 (82%)
Mean number of sex partners in the past month (SD)	2 (9)
Mean number of sex partners in the past month with whom no condom was used (SD)	1 (2)
Sexual behaviour at week 48	
Vaginal sex with a man in the past month	98 (75%)
Mean number of sex partners in the past month (SD)	2 (3)
Mean number of sex partners in the past month with whom no condom was used (SD)	1 (1)

^a^Participants self‐reported and were able to select more than one category. SD, standard deviation; HPTN, HIV Prevention Trials Network; ACTG, AIDS Clinical Trials Group.

As detailed in Table 1, the median age of female respondents who completed the parent trial on their assigned drug regimen (N = 130) was 39 years (range: 18‐61). Most were not married (90%), had completed high school but less than a four‐year college degree (82%) and were unemployed (59%). Almost three‐quarters of participants (74%) identified as non‐White and 13% self‐reported Hispanic/Latina ethnicity. At baseline, women reported an average of two sexual partners (SD: 9.0) in the past month and one partner with whom a condom was not used (SD: 2). Women completing the trial on study drug were largely comparable to those who discontinued early, with the exception of being older (39 vs. 32 years of age). Women in the interview subset did not significantly differ from the full sample of women in demography or behavioural characteristics.

### 3.2 Computer‐assisted self‐interview

#### 3.2.1 Self‐reported drug adherence

Participants reported generally high adherence at week 48. As presented in Table 2, more than three‐quarters of women reported taking study drug most of the time (35%) or always (44%) in the past 30 days. Most women similarly reported their ability to take daily study medications as very good (18%) or excellent (40%). Most women (75%) reported that they never over‐reported their adherence to study team members; 21% reported overestimating their actual adherence intentionally some to all of the time.

**Table 2:** Adherence to pre‐exposure prophylaxis study drugs reported by computer‐assisted self‐interview at week 48

	N = 130 (%)
Adherence in past 30 days	
All of the time	57 (44)
Most of the time	46 (35)
Half of the time	11 (7)
Some of the time	8 (6)
None of the time	3 (2)
Missing	5 (4)
Frequency of adherence over‐reporting	
Never	98 (75)
Some of the time	17 (13)
Most of the time	7 (5)
All of the time	4 (3)
Declined to answer	4 (3)
Ability to take study meds every day	
Excellent	51 (39)
Very good	23 (18)
Good	23 (18)
Fair	21 (16)
Poor	4 (3)
Very poor	2 (2)
Declined to answer	1 (1)
Missing	5 (4)

#### 3.2.2 Barriers and facilitators to product adherence

The most commonly cited challenges to adherence (Table 3) were “forgetting” (45%), being away from home (39%) and not having pills available when they were scheduled to be taken (22%). Few participants reported HIV stigma (4%), concern about disclosing participation in the study (4%) or concerns about side effects (8%).

**Table 3:** Barriers and facilitators for taking pre‐exposure prophylaxis during HPTN069/ACTG5305 study reported by women participants

	N = 130 (%)
Factors that made it difficult to take study medications^a^	
I did not have pills with me when I was supposed to take them	28 (22)
I got confused by the instructions for when to take the pill	1 (1)
I ran out of pills	2 (2)
I wasn't able to tell when sex was going to happen	3 (2)
I forgot	59 (45)
I was worried about others thinking I have HIV because they saw me taking the pill	5 (4)
I was worried about others knowing I was in the study because they saw me taking the pill	5 (4)
I did not have a private place to take the pill	2 (2)
I was worried about or experiencing side effects	10 (8)
I was away from home	50 (39)
Declined to answer	17 (13)
Factors that made it easier to take study medications	
I felt committed to finding a way to prevent HIV	82 (63)
I felt like it is my study	50 (38)
I kept pills available/with me when I would need them	36 (28)
I worked pill‐taking into something I do anyway	41 (32)
I got better/am good at planning for when I will and will not have sex	14 (11)
I used a reminder strategy	30 (23)
I (or the site) marked the days of the week on my pill case	17 (13)
I kept pills out where I could see them as a reminder	44 (34)
Other people have helped me stick to my regimen	11 (9)
I have had helpful conversations with study team members	16 (12)
I have had helpful conversations with other participants	4 (3)
I am scared of getting HIV	34 (26)
Other	8 (6)
Declined to answer	8 (5)

^a^Participants could select more than one response. HPTN, HIV Prevention Trials Network; ACTG, AIDS Clinical Trials Group.

Respondents identified commitment to preventing HIV infection (63%), a sense of “ownership” of the study (38%), and making sure pills are available (28%) and stored in a location that was easily visible to them (34%) as factors that made adherence easier (Table 3). About a third of participants integrated pill‐taking into existing routines (31%), developed reminder strategies (23%) or kept track of dosing on their pill boxes (13%). Twenty‐six per cent of women reported concern about HIV acquisition as facilitating adherence.

#### 3.2.3 Attitudes towards PrEP

Women estimated the efficacy of the study drugs (how good the study drugs were at preventing HIV infection), on average, to be 69% out of 100%. Most participants had an overall positive opinion of PrEP. As presented in Table 4, over 75% would recommend PrEP to others, and most (60%) believed PrEP would be good for “anyone.”

**Table 4:** Attitudes towards taking pre‐exposure prophylaxis (PrEP) during HIV Prevention Trials Network 069/AIDS Clinical Trials Group 5305 reported by women participants

	N = 130 (%)
How good are study meds at preventing HIV (0% to 100%)	
Mean (SD)	69 (28)
Would you recommend the PrEP medications used in this study to others	
Yes	98 (76)
No	1 (1)
Not sure	30 (23)
Declined to answer	1 (1)
Who would this kind of prevention approach be good for	
Anyone	78 (60)
Most people	32 (25)
Some people	12 (9)
Only very few people	6 (5)
Declined to answer	2 (1)
Since getting the study pills I do more to protect myself against HIV than I did before (valid N = 117)	
Agree	60 (48)
Somewhat agree	14 (11)
Neither agree nor disagree	19 (15)
Somewhat disagree	4 (3)
Disagree	27 (22)
Declined to answer	1 (1)
Which prevention strategies do you feel you have more personal control over	
Condoms (male or female)	65 (51)
Daily PrEP	25 (19)
Neither, same amount of control using either condoms or PrEP	37 (29)
Declined to answer	3 (2)

Almost half of participants (48%) reported an increase in HIV preventive behaviour while on study (“Since getting the study pills, I do more to protect myself against HIV than I did before”). In response to items asking about which prevention modalities women felt they had the most “control” over, half of women (50%) reported feeling they had more control over a partners’ condom use than daily PrEP; more than a quarter of women (29%) perceived an equal amount of control and only about 20% reported they felt more personal control over PrEP use.

### 3.3 In‐depth interviews (N = 26)

#### 3.3.1 Experience using study product

Two main themes were identified concerning women's experiences using study products: (1) unrealized anticipated side effects and (2) appreciation of the benefits of participating in a study. Women anticipated side effects over time, which were often not realized or dissipated within the first weeks of the study. A number of participants also described the importance of education and counselling by study staff in tempering their concerns about possible side effects

**Participant:** I had a little bit of diarrhea. That's it. No vomiting, no nausea, none of that. Just one day total. So it really wasn't bothersome at all in terms of, “I don't want to take this medicine because of it.”

**Participant:** At first, the study nurse said, “You might get some side‐effects,” and I was like, “Wait a minute. What am I getting myself into?” and then, once I started taking it and a week went by, I said, “Oh, okay, I see now,” and I would take my medicine every day, every day.

**Interviewer:** So you were a little nervous at first.

**Participant:** At first, yeah, because I didn't know how, because she said some people, it didn't make them feel right. I said, “Well, I wonder how it's going to affect me?”. She said, “It might not affect you the way it affect them,” so I started taking it, and I was fine. I was okay.

Participants also described experience with the study that were appreciated and described as beneficial, including regular HIV testing and health monitoring.

**Participant:** I liked the fact that I got checked for – I got my blood checked and everything, HIV tested.

**Participant:** I liked the fact that they gave you condoms. And even though they don't – I wouldn't – I didn't want to get paid for it but they was like “Well we pay you anyway.” I was like “Ok. That's fine.” I get the money, the bus tickets and then like I get the information. I get schooled on some things. I really like the fact that I could learn from it.

#### 3.3.2 Barriers and facilitators to product adherence

Four themes concerning barriers and facilitators to study drug adherence were identified (Table 5). Three themes described barriers to study product adherence: (1) the impact of competing demands (e.g. family, employment, unrelated health issues, unexpected events); (2) lack of established routine; and (3) believing one should not dose if the “prescribed” dose time was missed. Participants did not describe any concerns about side effects or perceived HIV‐related stigma as factors in non‐adherence.

**Table 5:** Barriers and facilitators to product adherence and illustrative quotes

Theme	Quotes
Barriers	
**Competing Demands** Discussion that supported this theme centred around obligations that took priority over participation in the research study, including family needs, employment, emergencies and other unplanned life events	**Participant:** Actually, I was actually stressed because my 18‐year‐old was locked up. So, I was running back and forth to court to see what they were going to do with him. But it took two months. So, out of the two months, I was just stressed because I was like, “I could lose my Section 8. I could be homeless.” And it was like I wasn't really focusing on this box right here [WISEPILL] because now I've got to fuck with my livelihood. You get what I'm saying? **Participant:** Well, I had to stop taking ‘em because I had missed my cycle… Once I missed my cycle, I already knew something wasn't right. So I was like, “Okay, I gotta be pregnant.” So I stopped. And the reason why I stopped, because I really didn't know if I was going to keep my baby or terminate my baby. **Participant:** I got hit by a van – during this study, and I didn't take my medicine because I was in the hospital, and Susana [study nurse] was like, “What's going on?” The phone was going and everything, so once I got out of the hospital, I came over here and let them know what happened, and I didn't take the medicine for, like, five days.
**Lack of Established Routine** Women discussed a lack of established routine, including changes in daily schedule, employment‐related events and long‐term travel or moving. Although these events often resulted in only one or two missed pills, some women reported that unanticipated events caused them to miss follow‐up clinic visits, leading to longer periods of non‐adherence	**Participant:** At the beginning when I had the little box [WISEPILL] with me. …But I think it's because I usually look at it and it was just like a box. So I need to remember the pills was in there – I don't know. But at the beginning was real hard for me to stay on track with taking the pills. But once the months went by and I got used to it, like, it was routine.” **Participant:** [I] just totally forgot because I'm not used to it…. I missed Tuesday, I missed Wednesday. That's when I said you know what, I've got to think of another way because I can't remember. I have a problem with memory so I need it to be in my sight. When I wake up, [yawns], there's the pill box: “Oh shit, there's my pills, got to take them.”
**Not Dosing if Dose Time was Missed** Some women reported not dosing when they missed their specific dose time, suggesting that some did not understand that they could take pre‐exposure prophylaxis any time during the day	**Participant:** Oh shit. I can't take them now it's after 12 o'clock.
Facilitators	
**Consciously Building Adherence Habits** Discussion that supported this theme centred around developing new routines and pill‐taking reminders. This included using technology (cell phones, alarm clocks), keeping pills in a visible location as well as developing routines for pill – taking individually or in conjunction with others	**Participant:** But you could set your timer on the phone, but when you're not used to taking pills, it's kinda hard. You forget. But once you start being in the study for a long time, you'll start taking them more regularly. **Participant:** I knew that I had to take them. So, when I get up I eat and I'll just take the pills – I knew I had to take it because I was – I'm in the research, so I knew I had to take it. So, I'd just wake up in the morning and eat and just take them. **Participant:** Yeah, and sometimes my fiancé said, “Did you take your medicine this morning?” and I was like, “Yes, I took it,” because he has diabetes so he takes his medicine, so I would take mine. He'd be like, “Take your medicine,” and I was like, “I'm taking it,” so he would take his medicine and I would take mine.

One overarching theme was identified as facilitating adherence: consciously building adherence habits (e.g. using cell phone alerts, keeping pills visible, engaging their social network to help with reminders). In addition, almost all of the women who were interviewed valued the care they received as research participants and expressed a desire to contribute to HIV prevention research, although participants did not link these factors to study drug adherence.

#### 3.3.3 Future use intentions

Of the twenty‐six women interviewed, only nine expressed clear intentions of using PrEP after the study ended. The most commonly cited reasons for not planning to use PrEP were (1) low perceived HIV risk and (2) uncertainty about PrEP access and efficacy (Table 6).

**Table 6:** Themes about pre‐exposure prophylaxis (PrEP) future use intentions, and illustrative quotes

Theme	Quotes
Low perceived HIV risk	**Participant:** I don't need them no more… I only have one partner and I've been with my partner for four years, so – it's a female. …When I was messing with men, I used condoms. **Participant:** Right now, no, just because I have one partner and he – I don't know. Maybe if I switched my life around a little bit but right now I don't think [so] just because I don't feel like my risk is very high. **Participant:** Yeah, I'd use it. I'm [in] an intimately, good relationship now, so I don't really foresee that changing. But, you know, you never know. And you never really know your partner's full history, I guess, unless you, you know, make them give them a medical report or something, so I'm pretty confident about the one I have now, but, you know, what could happen later you just don't – you can't predict.
Uncertainty about PrEP access and efficacy	**Interviewer:** Mm‐hmm. So it's [PrEP] something that you would use. So do you think you would use it by itself or part of another prevention… plan, so to speak? **Participant:** I think it would be the primary one. But I can also see, you know, using condoms. **Interviewer:** Okay. Okay. All right. And any idea as to where you might could get PrEP today? **Participant:** Well, I'm not sure if you can get it as PrEP… I don't think it's been approved for that yet. But hopefully, you know, after this it will be. **Participant:** That's a good question. I don't know. I … I don't think that I'm in like a risk bad enough for me to take it. And I'd also have to – I guess we didn't talk about – I never mentioned cost. I guess that would be a really good **Interviewer:** Part two of that question is do you know where you would go and get it, like your provider or – how much it costs or anything. **Participant:** No, I wouldn't even know how to start. So I guess that's also the facilitation, the question before, talking to your doctor about it, talking to your pharmacy about it, and then just having the money to actually do it. **Participant:** Yeah, as long as I could – my insurance would – but I know condoms the best way to prevent HIV. But it wouldn't be bad to take that pill. **Interviewer:** So if your insurance covered it, you think it would be something you would look into? **Participant**: Yeah. **Interviewer:** And you say you still would use condoms? **Participant:** Yes. **Interviewer:** Okay. Are you planning to use PrEP at some point in the near future? **Participant:** No. [Laughter] **Interviewer:** Why wouldn't you use PrEP? **Participant:** I mean I wouldn't even know how to get it… **Interviewer:** Like where to go, where to get it from? **Participant:** Yeah. Yeah. No idea. **Participant:** I think I would trust it. I mean depending on how the outcomes look and how effective it is. I mean I think that alone could be effective enough for preventing HIV if it's comparable to condoms or things like that. I mean total, I would also want a contraception or, you know, some way to prevent other STDs. So it would be in combination with other things too.

Women perceived themselves to be at low risk of HIV acquisition, citing their own monogamy, their partner's low risk of acquiring or having HIV, and condom use. A few women reported discontinuing sexual partnerships during the course of trial, and did not feel that they would need PrEP until they were once again sexually active. Valuation of risk appeared largely focused on women's own (vs. partner) behaviour, with being in a monogamous relationship often noted as evidence for no HIV risk.

Women also expressed uncertainty about where to access PrEP and whether it would be covered by insurance; some participants were unaware that oral PrEP with TDF/FTC was approved by the US Food and Drug Administration for at‐risk women in the US. Women were also unsure how PrEP would fit into their current HIV prevention strategies, whether it would be efficacious enough to replace condoms or whether it should be part of a combination of interventions for HIV prevention.

## 4 Discussion

Although most new HIV infections in the US are among men, more than 7000 women received an HIV diagnosis in 2016 1. We used mixed methods (CASI and IDIs) to explore acceptability of, adherence to, and intentions concerning future use of PrEP among US women who completed a 48‐week Phase II prospective, randomized, double‐blinded, multisite, safety and tolerability study of four candidate PrEP regimens comprised of combinations of TDF, FTC, and MVC. Women found the study regimens acceptable, but among those interviewed a lack of perceived risk or need and concerns about affordable access limited enthusiasm for using PrEP after the end of the study.

Women reported high levels of adherence, with most reporting taking their study drugs most or all of the time during the previous month; however, nearly a quarter of women acknowledged over‐reporting the proportion of study medications they had taken at times. Previous studies have linked intentional non‐adherence and over‐reporting adherence to study teams as related to concerns about safety and side effects 33, 34, 35, 36, 37; however, few women in our study cited side effects as barriers to adherence or discussed safety concerns in interviews. Participants discussed initial apprehension about side effects that were never realized, which may have decreased concerns about safety over time.

Lapses in drug adherence were often explained as unintentional and due to a lack of established routines, reminder tools and competing demands. Several women also reported the (erroneous) belief that a dose could or should not be taken if the exact “prescribed” dose time was missed. In CASI and interviews, facilitators for taking pills included developing habits, such as keeping pills visible, and a commitment to HIV research and to the study. The impact on adherence of developing habits and using reminder tools is well described in the ART (and other) adherence literature 38, 39, 40, 41, and further underscored by our findings. These results highlight the importance of verifying patients’ understanding about the timing of medications and what to do when doses are inadvertently missed or taken late.

Women had generally positive impressions of PrEP after participating in the study and believed PrEP would be a useful HIV prevention tool and “good for anyone,” with a majority indicating they would recommend it to others. However, some women in the interviews expressed uncertainty about using PrEP in the future because they were unsure about how PrEP would fit into their current prevention strategies and whether it would be accessible through insurance. In fact, several women seemed unaware that an FDA‐approved PrEP regimen was available to at‐risk women in the US. Intentions for PrEP uptake may have been higher if the study actively transitioned participants to PrEP prescribers in the local area. But the major reason for women's relative lack of interest in using PrEP after the study was their perception that they were not at risk for HIV acquisition. Discourse concerning participants’ valuation of risk was focused almost exclusively on the participant's behaviour – with little attention to the influence of partner characteristics and community HIV infection rates. Although women in our study may have accurately assessed their likelihood of acquiring HIV, their assessments could also have missed important external factors that elevated their risk, such as community‐level HIV risk. We do note that women, generally, did appear to report generally low HIV risk (condomless sex, number of partners) behaviours; however, future research should carefully consider associations between reported behaviour and valuation of HIV risk for women specifically. A more holistic discourse about HIV risk and risk perception that includes partner, community and structural influences may be particularly important for women's uptake of PrEP in communities that are highly impacted by HIV. Although our study was conducted in the US, this recommendation may generalize widely; women's unique vulnerabilities to HIV are worldwide 42 and campaigns that focus on individual risk factors may promote an underestimate of HIV risk for women.

Surprisingly, women did not appear to consider PrEP as a prevention strategy that was more under their control than condoms. Because condom use often requires women to negotiate with partners or otherwise rely on partner initiated behaviours and PrEP is self‐directed and can be administered without partner awareness 43, we had anticipated that most women would rate PrEP as more in their immediate control than condom use. The most commonly selected response to this item was perceiving more control over condom use. This may have been influenced by the study context – which involved taking a study drug under evaluation; where as they can visually see a condom being used and are confident in its function, they cannot see the study drug “working” and do not know if it is actually effective. Additionally, women were counselled on use of condoms throughout the study, with more than half reported that participation in the study increased their use of prevention strategies over time. Thus, this finding should be re‐examined in the context of open‐label (known to be effective), easily accessible, PrEP projects.

Findings should also be interpreted in the light of several limitations. First, only a small number of study participants at three sites were asked to participate in IDIs, thus may not be representative of the women in the overall study. Second, HPTN069/ACTG5305 trial participants were healthy volunteers in a blinded trial; women not engaging in research may differ from those who do in concerns about safety and challenges with intentional non‐adherence. Additionally, while eligibility criteria required participants to be at‐risk for HIV (i.e. condomless vaginal or anal intercourse with ≥1 HIV‐infected or unknown‐serostatus man within 90 days of enrolment), they were not necessarily at high risk for HIV infection. Finally, study drug adherence and related attitudes towards the drug and the regimen may dramatically differ from adherence and attitudes towards drugs known to be effective and widely available outside of a study context. Study participants completed interviews in late 2015. Efforts to increase PrEP awareness and access for women has increased in a number of regions in the US since that time, although PrEP uptake remains generally low. Nonetheless, women in our study may differ in terms of PrEP knowledge, awareness and attitudes from more contemporary samples 44.

Strengths of this study include a study population that is racially and ethnically similar to that of women living with HIV in the United States, with significant representation from Black and Hispanic women. Additionally, study sites were broadly representative of US major cities and geographically reflect US regions most affected by the HIV epidemic.

## 5 Conclusion

Women's valuation of their personal risk for HIV infection will be critical to PrEP's success as an HIV prevention strategy for women. Building women's demand for PrEP may be challenged by their personal risk assessments in settings where individual‐level risk factors appear minimal but contextual and partner‐level factors confer elevated risk for HIV. More holistic thinking about women's HIV risk will be needed to maximize the effectiveness of PrEP as an HIV prevention strategy for women in the US.

## Competing interests

Dr. Wilkin reports grants from Bristol‐Myers Squibb, Gilead Sciences and GlaxoSmithKline/ViiV Healthcare, and personal fees from GlaxoSmithKline/ViiV Healthcare outside the submitted work. Dr. Landovitz reports personal fees and nonfinancial support from Gilead Sciences outside the submitted work. Dr. Amico reports a grant from Gilead Sciences outside the submitted work. Dr. Swaminathan received research grants from Gilead Sciences and received honoraria for serving on advisory boards to Gilead Sciences. Dr. Hodder reports personal fees from Gilead Sciences, ViiV Healthcare, Janssen Pharmaceuticals and Bristol‐Myers Squibb outside the submitted work. The authors not named here have disclosed no conflicts of interest.

## Authors’ contributions

S.H., A.A.A. and K.R.A. designed the qualitative substudy. R.G., T.W. and K.H.M. chaired the main study's protocol, with K.R.A., R.J.L., S.H. and M.M. as protocol team members. S.S., S.H., A.A.A., K.R.A. and C.R. led the implementation of the qualitative substudy with N.D.T. implementing the interviews. J.W., M.R.C., B.E.M., J.S., C.R. and K.R.A. analysed the data. K.R.A. and C.R. wrote the manuscript with all other authors contributing on review and revisions.
